# Unraveling the role of coagulation-related genes in esophageal squamous cell carcinoma: development of a prognostic model and exploration of potential clinical significance

**DOI:** 10.3389/fonc.2025.1573279

**Published:** 2025-10-07

**Authors:** Langlang Deng, Chen Fang, Weiran Zhang, Zheng Zhu, Yu Gu, Pinchao Gu, Xiaoyan Tan, Jiamin Yuan, Yu Feng, Haitao Ma

**Affiliations:** ^1^ Department of Thoracic and Cardiovascular Surgery, The Fourth Affiliated Hospital of Soochow University, Suzhou, Jiangsu, China; ^2^ Department of Thoracic and Cardiovascular Surgery, Suqian First Hospital, Suqian, Jiangsu, China; ^3^ Department of Thoracic Surgery, The First Affiliated Hospital of Soochow University, Suzhou, Jiangsu, China; ^4^ Department of Cardiology, The First Affiliated Hospital of Soochow University, Suzhou, Jiangsu, China

**Keywords:** esophageal squamous cell carcinoma, coagulation, machine learning, RINT1, genomic instability, prognosis

## Abstract

**Background:**

Esophageal squamous cell carcinoma (ESCC), the most common form of esophageal cancer, is associated with high incidence and mortality rates, representing a major public health challenge. Although previous research has suggested a link between coagulation dysfunction and cancer progression, the precise role of coagulation-related genes in ESCC remains poorly understood.

**Methods:**

To investigate this, we integrated various multi-omics datasets, including mRNA expression data from TCGA and GEO, single-cell RNA sequencing data, as well as DNA mutation and methylation profiles. By applying machine learning algorithms, we identified coagulation-related genes in ESCC and developed a predictive model with clinical relevance. Further analyses were performed to assess the biological functions, prognostic significance, clinical implications, immune interactions, and drug sensitivity associated with these genes.

**Results:**

In this study, we identified seven coagulation feature genes—RAP1B, SRC, CFHR4, PLA2G4A, ORAI1, RINT1, and SPTB—in ESCC. A prognostic model based on these genes effectively stratified patients and demonstrated robust predictive value for clinical outcomes. Further analysis revealed distinct differences in immune function, drug sensitivity, and disease-related pathways between high- and low-risk groups. Among these genes, RINT1 emerged as a key factor, with pan-cancer analysis highlighting its potential relevance across multiple tumor types. We used immunohistochemistry, qRT-PCR, and Western blot to validate its differential expression in ESCC, highlighting its potential as a therapeutic target.

**Conclusion:**

Our findings emphasize the significance of coagulation-related genes in ESCC progression and their involvement in critical biological and immune processes. The proposed prognostic model provides a valuable tool for risk assessment. Additionally, the identification of RINT1 provides new insights as a potential prognostic biomarker and candidate for future therapeutic investigation in ESCC patients.

## 1 Introduction

Esophageal cancer (EC) is a major global health concern, ranking as the seventh leading cause of cancer-related mortality and the eleventh most common malignancy worldwide. In 2022, approximately 445,000 individuals worldwide succumbed to EC, with 511,000 new cases reported, underscoring its substantial disease burden (1). Although the global incidence of esophageal cancer has declined, a significant number of patients continue to be diagnosed at advanced stages, limiting treatment options and reducing survival rates (2). Despite advances in surgical techniques and adjuvant therapies, esophageal cancer remains associated with high rates of recurrence, metastasis, and drug resistance, which significantly complicate patient management (3). Notably, esophageal squamous cell carcinoma (ESCC) accounts for nearly 80% of all esophageal cancer cases, particularly in low-income regions, where it poses a critical public health challenge (4). Addressing these issues requires a deeper understanding of ESCC pathogenesis and the exploration of innovative therapeutic strategies.

Dysregulation of coagulation is increasingly recognized as a key factor in cancer progression, contributing to both thrombotic and hemorrhagic complications in affected patients. Studies indicate that hypercoagulability-related venous thromboembolism (VTE) accounts for approximately 20% of all VTE cases in oncology patients (5, 6), and cancer-associated thrombosis (CAT) was historically considered the second leading cause of cancer-related mortality (7). Moreover, due to coagulation abnormalities and direct tumor invasion, advanced malignancies such as lung and gastric cancers frequently present with hemorrhagic symptoms (8, 9), Similar hemostatic challenges have been observed in ESCC, highlighting the need for further investigation into the interplay between coagulation dysfunction and tumor biology (10). Research indicates that coagulation pathways play crucial roles in cancer progression. Thrombotic complications may indicate occult cancers (11), and the activation of the coagulation cascade plays a key role in this process. Specifically, tumor cells promote the manufacture or secretion of different chemicals to affect platelet function, hence encouraging tumor cell proliferation and metastasis (12). High expression of tissue factor (TF) and thrombin intensifies the hypercoagulable state, drug resistance, and metastasis of cancer through specific pathways, often correlating with poor prognosis (13, 14). Fibrinogen, as the end product of the cascade, not only promotes tumor cell migration but also shields tumor cells from immune surveillance (15). Furthermore, coagulation pathways are involved in tumor inflammation and immune responses. Proinflammatory factors released by platelets not only recruit and activate leukocytes but also exacerbate the pathogenesis of diseases such as cancer (16). Coagulation proteases enhance cancer immune evasion by driving specific pathways, thereby supporting tumor growth and development (17). According to pan-cancer research, coagulation pathways are closely related to the expression of immunological checkpoints and the tumor microenvironment (18). Consequently, there is great research value in coagulation-related genes for tumor diagnosis and treatment.

The use of bioinformatics in cancer research has greatly progressed in recent years, with new methods offering vital assistance (19). Large volumes of genomic, transcriptomic, epigenomic, and proteomic data have been produced by high-throughput sequencing technology and computational biology techniques. Through comprehensive analysis of multi-omics data, researchers can identify unique molecular features, enabling personalized treatment plans (20, 21). The development of many cancer therapies is closely linked to bioinformatics, such as the successful use of immune checkpoint inhibitors in cancer immunotherapy, which benefits from in-depth analysis of the immune microenvironment (IME) (22, 23). As artificial intelligence advances, various neural network and machine learning algorithms are being employed to integrate multi-omics data (24), revealing the complex molecular mechanisms of cancer. Algorithms such as elastic net and random forest are utilized for feature selection and model construction, demonstrating significant value in early cancer diagnosis, personalized therapy, prognosis assessment, and the development of novel treatments (25). These bioinformatics techniques enhance the quality of life and survival rates of cancer patients by aiding in the development of novel treatments and clinical translation (26). Genomic instability is a central phenomenon in cancer biology, underpinning tumor initiation, progression, drug resistance, and treatment (27). Investigating genomic instability not only uncovers fundamental mechanisms of tumorigenesis but also provides a critical foundation for prognostic evaluation, predicting treatment sensitivity, and identifying potential therapeutic targets. Bioinformatics approaches are increasingly crucial in assessing the role of key genes in genomic instability (28).

Previous research has demonstrated the unique roles and prognostic value of several coagulation biomarkers (e.g., PLT, MPV, fibrinogen, thrombin time, thrombin receptor, and tissue factor) in ESCC (29–31). However, the potential regulatory role of coagulation-related genes in ESCC remains unexplored. Further investigation into the relationship between these genes and ESCC is essential to identify novel biomarkers for diagnosis and treatment, providing new support for personalized therapy. In order to thoroughly examine the functions of coagulation-related genes in ESCC, we combined mRNA expression levels, single-cell RNA sequencing, DNA mutations, and methylation data. Using a combination of machine learning algorithms, we identified coagulation feature genes in ESCC and build a prognostic model based on these findings to predict personalized treatment outcomes for ESCC patients. Through *in vitro* experiments and pan-cancer research, we investigated the function of the important coagulation gene RINT1 and further evaluated the significance of coagulation feature genes in ESCC using this model.

## 2 Materials and methods

### 2.1 Data collection and preliminary processing

Gene expression and clinical data for ESCC patients were obtained from The Cancer Genome Atlas (TCGA, https://portal.gdc.cancer.gov/) and the Gene Expression Omnibus (GEO, https://www.ncbi.nlm.nih.gov/geo/) databases. Specifically, 94 ESCC samples were obtained from the TCGA-ESCA cohort, and 60 and 119 ESCC samples were acquired from two GEO datasets (GSE53622 and GSE53624). These datasets were subjected to batch effect correction using the “combat” approach, which produced a gene expression dataset that included 273 tumor samples. Mutation and copy number variation data were obtained from the TCGA database. Coagulation-related gene data were sourced from the GSEA website (www.gsea-msigdb.org) and the KEGG website (https://www.kegg.jp). Using “coagulation” as the keyword, we reviewed the search results and selected the biological pathways most closely related to the coagulation cascade; a total of 10 coagulation-related pathways were identified and included. Ultimately, 480 coagulation-related genes were extracted. **Supplementary Table S1** provides information on the genes and pathways.

### 2.2 Genomic variation and survival analysis

TCGA sample visualization and genomic mutation analysis were performed using the “maftools” software. CNV analysis was performed to investigate DNA segment amplifications or deletions, which are important in various diseases, including cancer. TCGA-derived CNV data were analyzed with R. Kaplan-Meier survival analysis and univariate Cox regression were applied to examine the survival effects of coagulation-related genes and identify significant genes for further study.

### 2.3 Consensus clustering analysis of coagulation-related genes and ESCC samples

Using survival data from ESCC samples and the expression data of 27 coagulation-related genes with prognostic significance found by univariate Cox regression analysis, we used the “ConsensusClusterPlus” program to conduct consistency clustering analysis. The “Partitioning Around Medoids (PAM)” method was employed with a sampling rate of 0.8 for each iteration, and 100 iterations were performed to ensure stable clustering results (32). The final analysis identified two clusters for subsequent analysis.

### 2.4 Gene set variation analysis, functional enrichment analysis, and immune microenvironment analysis

Various methods were employed to analyze the functional roles of coagulation-related genes under different conditions. Gene Set Variation Analysis (GSVA), an unsupervised approach, assessed the enrichment levels of coagulation-related gene sets across samples (33). Gene Set Enrichment Analysis (GSEA), a supervised method, determined the significant enrichment of predefined gene sets between different phenotypes, further comparing enrichment under different conditions using GSEA. Single-sample Gene Set Enrichment Analysis (ssGSEA) was used to generate immune cell scores for each sample, allowing for differential analysis of immune cell populations (34). The CIBERSORT (Cell-type Identification by Estimating Relative Subsets of RNA Transcripts) algorithm analyzed immune cell infiltration in the ESCC microenvironment. These methods collectively provided a comprehensive characterization of the IME. The “estimate” package analyzed the ESCC microenvironment to determine relative abundances of different cell types, elucidating mechanisms underlying tumor development and treatment responses. GO and KEGG enrichment analyses elucidated the functions and pathway associations of the coagulation gene set, revealing biological processes and mechanisms relevant to coagulation genes in ESCC and their potential value in research.

### 2.5 Establishment of machine learning and clinical prognosis models

The samples were divided into training (137 ESCC samples) and testing (136 ESCC samples) groups using a 50% split ratio (“p = 0.5”), with the process repeated 1000 times. We utilized 101 combinations of machine learning algorithms for prognostic feature selection. These algorithms were sourced from a professional online platform (http://www.sxdyc.com/). After systematic screening, 97 algorithms were selected for further analysis, and their respective C-index values were calculated. Genes included in the model with the highest average C-index were subsequently chosen for downstream analysis. The RSF algorithm demonstrated the highest C-index among all models. It was implemented using 1000 trees, with a nodesize parameter of 5, and variable importance was evaluated for each feature. Multi-factor Cox regression analysis employed the “both” method to further screen feature genes, obtaining risk coefficients for each. Subsequently, the “predict” function calculated sample risk scores using the formula:


$$Riskscore=\;h_{0\;}\left(t\right)*\text{exp}\left(\beta_{1}X_{1}+\beta_{2}X_{2}+\cdots +\beta_{n}X_{n}\right)$$

Where $h_{0\;}\left(t\right)$ represents the baseline hazard function indicating the basic probability of an event occurring without other influencing factors, and $\beta_{1}$ , $\beta_{2}$ , …, $\beta_{n}$ , are the risk coefficients obtained from model fitting, $X_{1}$ , $X_{2}$ ,… $X_{n}$ represent the expression levels of corresponding coagulation feature genes. Seven coagulation feature genes and their respective risk coefficients were ultimately identified.

### 2.6 Model evaluation and drug sensitivity analysis

The prognostic value of the established model was evaluated using a number of R packages, such as the “survival,” “timeROC,” and “rms” packages, which evaluated survival prediction by plotting survival curves, receiver operating characteristic (ROC) curves, and calculating the area under the ROC curve (AUC); single-factor and multi-factor Cox regression analyses were used to assess the association between risk scores, clinical features, and prognosis (35). Various plots, such as nomogram, calibration curves, and decision curves, were generated using data from these packages. Additionally, the “oncoPredict” package was used to determine the sensitivity of various risk score groups to chemotherapy drugs commonly used in cancer treatment. This analysis provided potential pharmaceutical support for personalized therapy in ESCC (36).

### 2.7 single-cell analysis

Single-cell data from the GSE160269 and GSE173950 cohorts were analyzed using the Tumor Immune Single-cell Hub 2 (TISCH2, http://tisch.comp-genomics.org/), allowing for a preliminary exploration of the potential roles of coagulation-related genes in esophageal cancer.

To gain deeper insights and perform a more refined analysis, we selected single-cell data from three ESCC tumor samples and adjacent normal tissues of patients from the GSE196756 dataset. Data analysis was performed using Seurat (version 5.1.0) (37). To ensure data quality, we applied the following criteria: nCount_RNA ≥ 1000, nFeature_RNA ≥ 200 and ≤ 10,000, mitochondrial gene percentage ≤ 25%, and ribosomal gene percentage > 3%. After removing batch effects using the Harmony method, we performed LogNormalize standardization and identified the top 2000 highly variable genes for subsequent analysis. Dimensionality reduction was performed using PCA, followed by clustering with the “FindClusters” function. Based on the clustering visualization, we selected a resolution of 0.8, identifying 21 distinct clusters. Subgroup clustering was conducted using classical ESCC marker genes.

We performed differential expression analysis using data from the GSE53622 and GSE53624 datasets, to distinguish the gene expression profiles of tumor tissues from those of normal tissues. The top 50 significantly upregulated genes in tumor tissues were defined as the gene set for calculating the malignancy score, while the top 50 significantly downregulated genes were used to compute the non-malignancy score. We used the UCell package’s “AddModuleScore_UCell” function to calculate malignancy and non-malignancy scores for each cell. Finally, epithelial cells with high malignancy scores and low non-malignancy scores were identified as malignant epithelial cells. We identified genes associated with epithelial-mesenchymal transition (EMT) from existing literature, including VIM, SNAI1, MMP9, AREG, SERPINH1, and FAT1, and used the “AddModuleScore_UCell” method to calculate EMT and coagulation scores, comparing the trends in their correlation. We also performed pseudotime analysis on malignant epithelial cells using the “monocle2” tool. We further analyzed the immune cell subpopulations in ESCC based on classical marker genes and used the “singleR” method to distinguish T/NK cell subpopulations. Additionally, we examined the expression of key coagulation genes in immune cells.

### 2.8 Pan-cancer analysis

To identify key coagulation genes, we established a protein-protein interaction (PPI) network via the STRING database (https://cn.string-db.org/) (38). The resulting analysis was imported into Cytoscape for further exploration, where significant genes were filtered, and the “psych” package was utilized to study gene correlations. An online analysis platform (http://www.sxdyc.com/) was used to do a pan-cancer analysis of the coagulation gene RINT1, which provided RINT1 expression data and immune infiltration analysis from TCGA, GTEx, and other databases. This platform offers professional analysis and visualizations of immune-related data. For integrated gene set cancer analysis, we utilized the GSCA website (https://guolab.wchscu.cn/GSCA), which includes pan-cancer CNV and methylation data. This made it possible for us to investigate the connections among various cancer types between gene expression, DNA methylation levels, and gene mutations.

### 2.9 Transcription factor and RBP analysis

We used the KnockTF 2.0 database (http://www.licpathway.net/KnockTF/index.php) to analyze potential transcription factors of key coagulation genes (39). To explore potential RNA-binding proteins (RBPs) of key coagulation genes, we utilized the ENCORI database (https://rnasysu.com/encori/). These analyses provide insights into the potential role of coagulation key genes in post-transcriptional regulation (40).

### 2.10 Cell lines and reagents

Human ESCC cell lines KYSE-30, KYSE-150, and normal esophageal epithelial cells HET-1A were purchased from EallBio. Polyclonal antibodies against RINT1 and GAPDH were purchased from Proteintech. HRP-conjugated goat anti-rabbit IgG and HRP-conjugated goat anti-mouse IgG were obtained from NCM Biotech. The Cell Total RNA Isolation Kit, All-in-One RT SuperMix, and SYBR Master Mix used for PCR experiments were acquired from Vazyme.

### 2.11 Cell culture conditions

The cell lines were grown in RPMI 1640 medium containing 10% fetal bovine serum and incubated at 37°C with 5% CO2 in a humidified incubator.

### 2.12 Immunohistochemistry

Patients from Soochow University’s First Affiliated Hospital provided tissue samples for ESCC. All patients underwent surgical treatment and were diagnosed with ESCC via postoperative pathology. Tumor and adjacent non-tumorous esophageal tissues, confirmed by pathological examination, were collected as paired samples; the normal tissues served as positive controls in the immunohistochemical analysis. Tissue sections were dewaxed in xylene, rehydrated through a graded ethanol series, and washed with water. Antigen retrieval was carried out by heating the sections in sodium citrate buffer, followed by natural cooling. To prevent nonspecific binding, sections were blocked with 3% hydrogen peroxide at room temperature. Primary antibodies (1:200 dilution) were incubated overnight at 4°C, followed by secondary antibody incubation (1:200 dilution) at room temperature for 1 hour. DAB staining and hematoxylin counterstaining were used for protein visualization. After washing, sections were mounted and examined under a microscope for imaging.

### 2.13 qRT-PCR

Using a R RNA extraction kit and the manufacturer’s instructions, total RNA was extracted from the three cell lines. A cDNA synthesis reagent was then used to reverse-transcribe the RNA into cDNA. The 2−ΔΔCt technique was used to quantify relative gene expression, and each sample was examined three times to ensure accurate findings. The primers listed below were employed for quantitative real-time PCR (qRT-PCR): RINT1 forward: GGCTGGGTAGTGAGTGTGTC, RINT1 reverse: ACTTTCAGAGCAGCACGGG, GAPDH forward: GCACCGTCAAGGCTGAGAAC, GAPDH reverse: TGGTGAAGACGCCAGTGGA.

### 2.14 Western blot

RIPA buffer enhanced with PMSF and phosphatase inhibitors was used to lyse the cells. After separation by SDS-PAGE, the proteins were transferred to PVDF membranes through electroblotting. To block nonspecific binding, membranes were incubated with 5% skim milk at room temperature for 1 hour. The target protein was detected by incubating the membranes overnight at 4°C with primary antibodies (1:2000 dilution). Following this, secondary antibodies (1:2000 dilution) were applied at room temperature for 1–2 hours. Protein bands were visualized using ECL chemiluminescence and analyzed with a chemiluminescent imaging system. Band intensity was quantified with ImageJ software, and GAPDH served as the internal control. Each experiment was performed in triplicate.

### 2.15 Statistical analysis

Statistical analysis was performed using R software (version 4.3.3). For differential analysis, the Wilcoxon test was used for data that did not follow a normal distribution, while the independent sample t-test was applied to normally distributed data. Spearman and Pearson correlation analyses were conducted to examine the relationship between gene expression and clinical features. In survival analysis, Kaplan-Meier survival curves, along with univariate and multivariate Cox regression, were employed to explore the association between genes and patient prognosis. Model evaluation was carried out using the “survival,” “timeROC,” and “rms” packages in R, generating survival curves, ROC curves, nomograms, calibration plots, and decision curves. Statistical significance was set at a p-value < 0.05.

## 3 Results

### 3.1 Mutational landscape and prognostic significance of coagulation-related genes in ESCC

**Figure 1:**
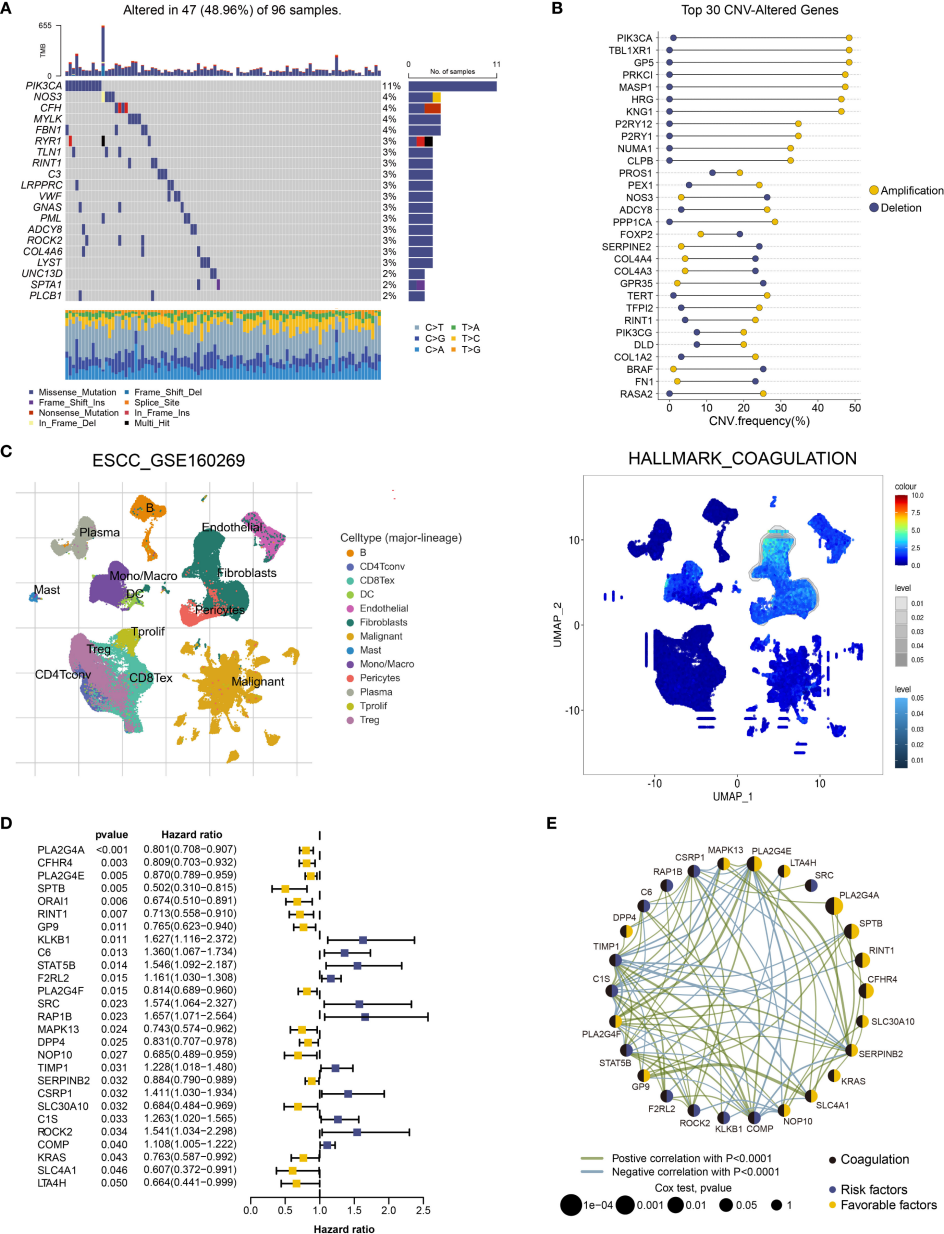
Functional landscape of coagulation-related genes in ESCC. **(A)** Oncoplot of somatic mutations in coagulation-related genes in the TCGA-ESCA cohort for ESCC.**(B)** Dumbbell plot of the top 30 coagulation-related genes with CNV alterations in ESCC. **(C)** Enrichment Scores of the Coagulation Pathway Across Different Cell Types in the GSE160269 Dataset Using GSEA. **(D)** Twenty-seven coagulation-related genes exhibiting significant prognostic capability in univariate Cox regression analysis. **(E)** Prognostic network of coagulation-related genes with significant prognostic impact.

We retrieved comprehensive genomic mutation data for ESCC from the TCGA database and selected mutation data specific to coagulation-related genes for further analysis. The results revealed that, compared to the comprehensive genomic dataset, coagulation-related genes exhibited a lower mutation frequency in ESCC, with missense mutations being the predominant type (**Figure 1A**, **Supplementary Figure S1A**). Additionally, coagulation-related genes exhibited a high frequency of copy number variations (CNVs), with amplifications and deletions occurring at similar rates (**Figure 1B**, **Supplementary Figure S1B**). Higher CNV frequencies are generally associated with genomic instability, suggesting that tumor cells may gain advantages in processes such as proliferation, adaptation, and immune evasion. Thus, coagulation-related genes may contribute to ESCC development through these mechanisms.

To further investigate the potential roles of coagulation-related genes in esophageal cancer, we analyzed single-cell datasets from different pathological subtypes: GSE160269 (esophageal squamous cell carcinoma, ESCC) and GSE173950 (esophageal adenocarcinoma, EAC). The analysis revealed that fibroblasts, endothelial cells, and myofibroblasts exhibited higher enrichment scores in coagulation-related pathways (**Figure 1C**, **Supplementary Figure S1C**). This finding suggests that coagulation-related genes may regulate the functions of these cell types, influencing processes such as angiogenesis, matrix remodeling, and immune modulation, thereby contributing to the regulation of the esophageal cancer microenvironment. Therefore, coagulation-related genes may play a critical role in the initiation and progression of esophageal cancer. Based on these observations, we conducted a more in-depth investigation of the roles of coagulation-related genes in ESCC.

We collected ESCC data from the TCGA and GEO databases, including TCGA-ESCA (94 samples), GSE53622 (60 samples), and GSE53624 (119 samples). After batch effect correction, we combined these datasets into one containing 273 ESCC samples, all of which had survival information and expression data for 31,366 genes. We conducted a prognostic analysis of coagulation-related genes using univariate Cox regression and Kaplan-Meier survival analysis on this integrated dataset, with statistical significance defined as p < 0.05. A total of 27 coagulation-related genes were identified as significantly associated with prognosis. Forest plots and prognostic network diagrams illustrated the prognostic value of these genes (**Figures 1D, E**). These analyses suggest that coagulation-related genes are crucial in ESCC prognosis and may impact clinical outcomes through specific mechanisms.

### 3.2 Identification of ESCC subtypes based on prognostic coagulation-related genes

**Figure 2:**
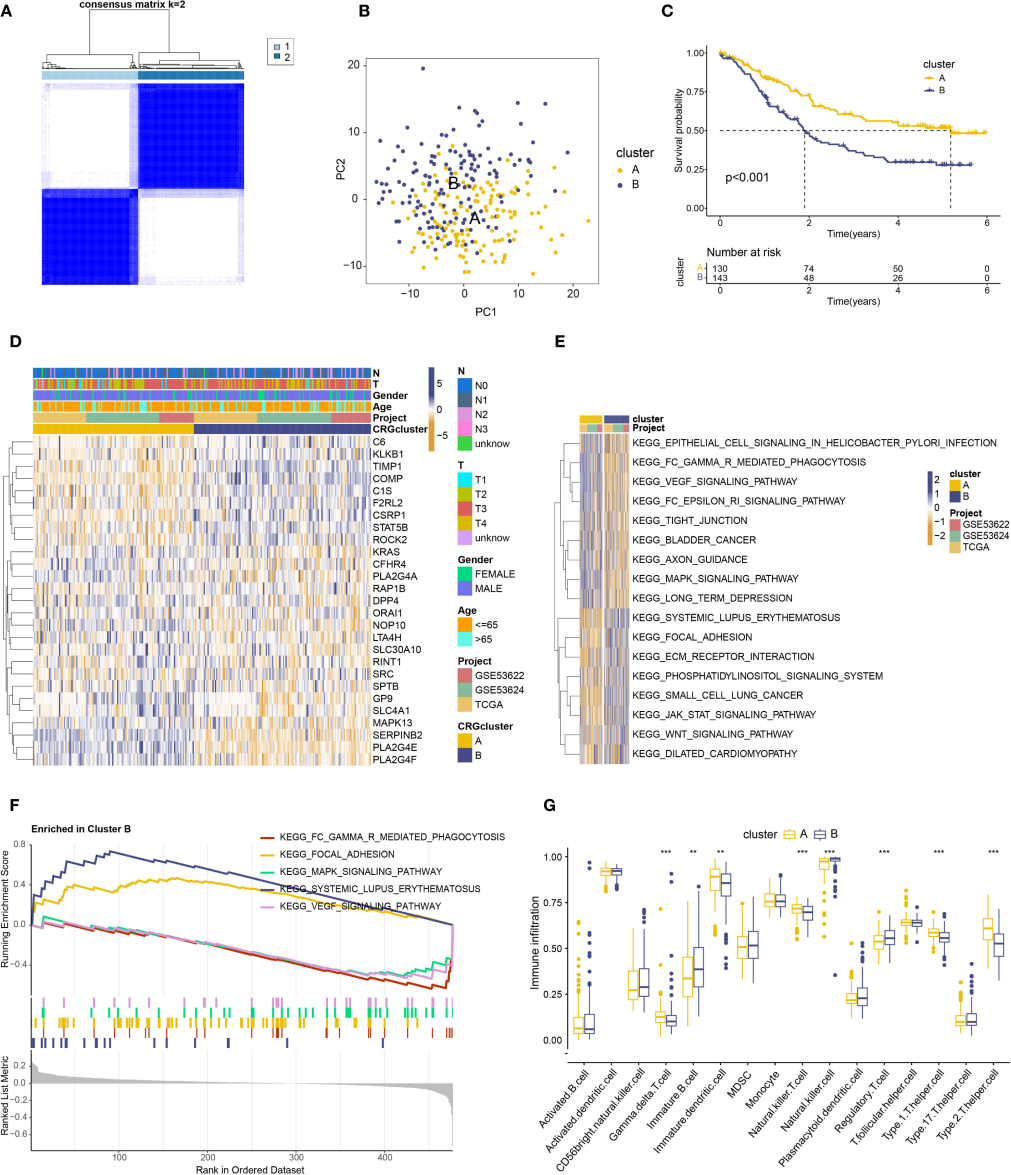
Prognostic analysis of coagulation-related genes in ESCC. **(A)** Two identified subtypes from consensus clustering**(B)** PCA analysis of the identified subtypes. **(C)** Kaplan–Meier analysis of the two subtypes. **(D)** Heatmap showing the relationship between the two subtypes, gene expression, and clinical features. **(E)** Heatmap of GSVA analysis for the two subtypes. **(F)** Significant pathway map from GSEA analysis of subtype **B. (G)** Box plot showing immune cell differences between the two subtypes based on ssGSEA analysis. ** : p-value < 0.01; *** : p-value < 0.001.

To gain deeper insight into the expression patterns and prognostic impact of coagulation-related genes in ESCC, we analyzed the expression profiles of 27 genes previously identified as prognostically significant. Using the “ConsensusClusterPlus” package in R, we applied consensus clustering to ESCC samples, which led to the identification of two distinct molecular subtypes (**Figure 2A**, **Supplementary Figure S1D**). The separation between these subtypes was further supported by principal component analysis (PCA), where the clustering pattern appeared to be well-defined (**Figure 2B**).

Next, we explored the clinical implications of these subtypes. Survival analysis suggested that patients classified as Subtype A tended to have a more favorable prognosis compared to those in Subtype B (**Figure 2C**). We also compiled clinical data, including age, sex, T stage, and N stage, and visualized these parameters in a heatmap (**Figure 2D**). Notably, patients in Subtype B were, on average, older and exhibited a higher prevalence of advanced T and N stages, suggesting a potential association between disease progression and subtype classification.

Taken together, these findings indicate that ESCC can be categorized into two molecularly distinct subtypes based on coagulation-related gene expression. While the precise biological implications of these differences require further investigation, our results suggest that coagulation-related genes may play an important role in disease stratification and prognosis prediction in ESCC.

### 3.3 Differential expression and function roles of coagulation-related genes in ESCC subtypes

To better understand the potential role of coagulation-related genes in ESCC progression, we examined differential gene expression patterns and performed functional enrichment analyses for the newly defined ESCC subtypes. Using GSVA, we found that subtype B was highly enriched in pathways related to immune function and tumor progression, including systemic lupus erythematosus pathogenesis, focal adhesion, JAK-STAT signaling, phosphoinositide signaling, and Wnt signaling (**Figure 2E**). The prominence of these pathways suggests that coagulation-related genes in subtype B could be involved in modulating immune responses, inflammatory processes, and key signaling cascades that drive tumor development. However, further investigation is needed to clarify the precise molecular mechanisms underlying these associations.

Further GSEA analysis (**Figure 2F**) revealed a significant enrichment of subtype B in pathways related to systemic lupus erythematosus and focal adhesion, potentially reflecting underlying immune dysregulation and metastatic behavior. Notably, these pathways have been implicated in immune evasion and tumor progression, suggesting that subtype B may exhibit a more aggressive phenotype. While this observation may partly explain its poor prognosis, further research is required to substantiate these findings. Additionally, whether these molecular characteristics influence responsiveness to immunotherapy or targeted treatments remains an open question and warrants further investigation.

GSVA and GSEA analyses revealed that coagulation-related genes in subtype B are crucial for immune regulation, suggesting a need for further exploration of the relationship between coagulation genes and immune modulation. Immune cell differential analysis using ssGSEA (**Figure 2G**) revealed significantly higher expression of immature B cells, NK cells, and regulatory T cells in Subtype B, while Subtype A showed higher expression of γδ T cells, immature dendritic cells, NKT cells, Th1 cells, and Th2 cells.

We also performed differential analysis of the ESCC dataset using the “limma” package in R, stratified by the two subtypes, and identified 300 differentially expressed genes with |logFC| > 1 and adj.P.Val < 0.05. Visualization through a volcano plot revealed distinct expression patterns (**Supplementary Figure S1E**). Subsequent GO and KEGG analyses indicated their involvement in epidermal and keratinocyte differentiation pathways (GO analysis) and significant enrichment in inflammation, immune regulation, and signal transduction processes (KEGG analysis)(**Supplementary Figures S2A, B**). These findings align with existing research and deepen our understanding of functional and expression differences in clot-related genes in ESCC.

In conclusion, these analyses highlight potential differences in the function and expression of coagulation-related genes in ESCC, providing critical insights for further exploration of their roles in this disease.

### 3.4 Identification of coagulation feature genes and construction of a clinical prognostic model using integrated machine learning algorithms

**Figure 3:**
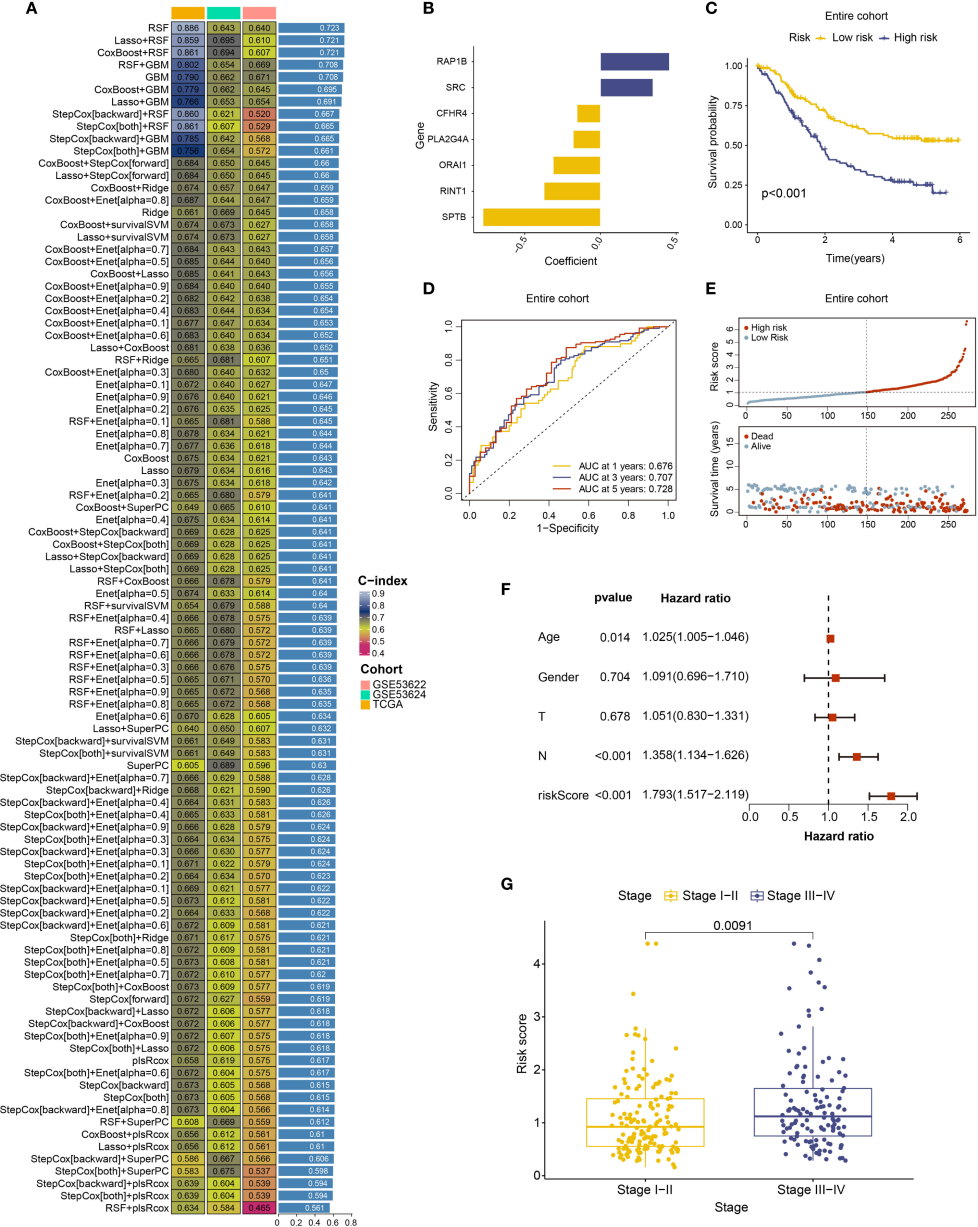
Machine learning algorithms identifying coagulation-related genes and establishing prediction models. **(A)** C-index ranking plot from analysis using 101 machine learning algorithms. **(B)** Risk coefficient plot of 7 coagulation feature genes. **(C)** Survival analysis of high-risk and low-risk patient groups in the entire cohort. **(D)** ROC curves and corresponding AUC values analyzed for patients stratified into high- and low-risk groups in the entire cohort at 1, 3, and 5 years. **(E)** Risk curves for high-risk and low-risk patient groups in the entire cohort. **(F)** Multivariate Cox regression analysis of risk scores with age, gender, T stage, and N stage. **(G)** Relationship between risk scores and clinical staging of ESCC.

Building on prior analyses indicating the potential prognostic value of coagulation-related genes in ESCC, The ESCC samples underwent gene selection utilizing 101 machine learning algorithms after being split into training and testing groups at random in a 1:1 ratio. The results (**Figure 3A**) showed that the RSF algorithm exhibited the highest average c-index (0.723) and successfully identified nine significant coagulation-related genes. To further evaluate the independent prognostic impact of each gene, calculate their risk coefficients, and mitigate model overfitting, we applied multivariate Cox regression analysis to quantify and assess the prognostic relevance of the genes selected by Random Survival Forest (RSF). Ultimately, we identified seven coagulation feature genes and their corresponding risk coefficients: CFHR4 (-0.15489), RINT1 (-0.37298), SPTB (-0.77620), PLA2G4A (-0.18017), SRC (0.34724), ORAI1 (-0.31232), and RAP1B (0.45536). **Figure 3B** shows a risk coefficient plot with the risk coefficients for each gene. The results indicated that worse clinical outcomes in ESCC were linked to higher expression of RAP1B and SRC, while high expression of CFHR4, PLA2G4A, ORAI1, RINT1, and SPTB correlated with better clinical outcomes. We also developed a prognostic model for ESCC and calculated the risk score for each sample based on coagulation feature genes. Based on the median risk score, we separated the samples into high-risk and low-risk groups for additional study.

We plotted survival curves and receiver operating characteristic (ROC) curves using the prognostic model that was created. The model performed well in both the training and testing groups, as well as throughout the full cohort, according to the result. The survival curve including all patients showed that the high-risk group had a significantly worse prognosis compared to the low-risk group (**Figure 3C**), and the survival curves of the training and testing groups also showed a consistent trend (**Supplementary Figure S2C**). The ROC curve and area under the curve (AUC) showed that the 3-year and 5-year AUC values for the cohort were both greater than 0.7, with the AUC improving over time (**Figure 3D**, **Supplementary Figure S2D**). we constructed a risk curve for the prognostic model, which revealed that as the risk score increased, the mortality rate also progressively rose (**Figure 3E**, **Supplementary Figure S2E**).

In summary, we successfully identified coagulation feature genes and developed a prognostic model, which exhibited good predictive performance. A higher risk score was associated with poorer clinical outcomes. This coagulation-related gene-based prognostic model offers a novel research perspective for ESCC treatment.

### 3.5 Clinical characteristics and immune microenvironment analysis of the coagulation feature gene based prognostic model

Using the ESCC coagulation gene-related prognostic model and patient data classified into high and low-risk groups, we performed an additional analysis incorporating clinical information. First, a forest plot of prognostic factors was created using both univariate and multivariate Cox regression, and independent prognostic analysis was carried out using the “survival” software. The findings showed that age, N stage, and risk score were independent predictors of ESCC (**Figure 3F**, **Supplementary Figure S3A**), while gender and T stage did not show statistical significance. Based on clinical staging, samples were then divided into low-stage (Stage I–II) and high-stage (Stage III–IV) groups. Using the “limma” program, a differential analysis of risk score among these groups was carried out. The findings suggest that higher clinical stages are generally associated with higher risk scores (**Figure 3G**). Additionally, our research indicated that subtype B exhibited a significantly higher risk score compared to other subtypes (**Supplementary Figure S3B**) and a poorer prognosis, which supports the predictive capacity of the prognostic model.

**Figure 4:**
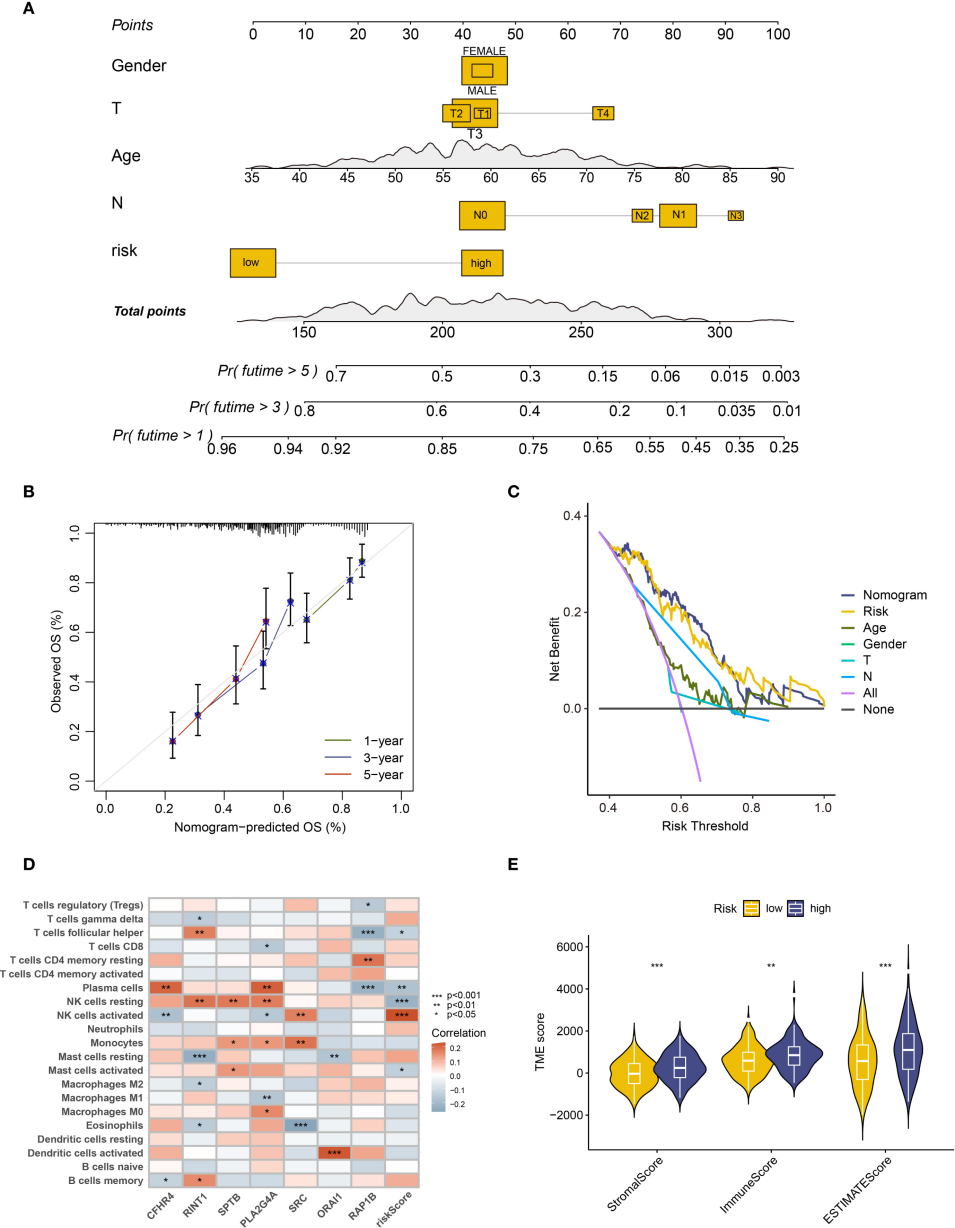
Clinical feature analysis and immune microenvironment analysis of prognostic models. **(A)** The nomogram constructed based on gender, age, T stage, N stage, and risk grouping. **(B)** Calibration curve of the column chart. **(C)** Decision curve analysis (DCA) at 5 years. **(D)** Heatmap showing the correlation between risk scores and immune cells. **(E)** Analysis of microenvironment differences in high and low-risk group patients. ** : p-value < 0.01; *** : p-value < 0.001.

To further assess the predictive ability of the prognostic model, we developed a nomogram using the available clinical data (**Figure 4A**). This nomogram, which incorporates patient gender, age, T stage, N stage, and risk score, offers a visual estimation of 1-, 3-, and 5-year survival probabilities. The cumulative risk curve (**Supplementary Figure S3C**) showed that, over time, high-risk patients in the nomogram had a higher prognostic risk. We plotted calibration curves for 1-, 3-, and 5-year survival in order to verify the nomogram’s prediction accuracy. The results suggest good predictive performance, with the calibration curves approaching the diagonal, which is indicative of better model accuracy (**Figure 4B**). To evaluate the model’s sensitivity and specificity, decision curves (DCA) were plotted at various intervals. The outcomes suggest that the model’s performance improved over time (**Figure 4C**, **Supplementary Figure S3D**). These analyses provide clinicians with an intuitive tool to better understand the clinical utility of the predictive model, facilitating its application in real-world treatment settings.

Furthermore, we applied the prognostic model and risk stratification to conduct an IME analysis, aiming to explore the complex effects of coagulation-related genes on ESCC patients. First, the Cibersort algorithm examined the differences in immune infiltration between high- and low-risk groups. The findings revealed variations between natural killer cells (NK cells) and activated mast cells (**Supplementary Figure S3E**). Moreover, a gene-risk score association heatmap showed that a number of immune cell types, such as resting and active NK cells, activated mast cells, follicular helper T cells, and plasma cells, showed a substantial correlation with risk score (**Figure 4D**). Among these, the expression of the majority of immune cells significantly associated with risk scores, with the exception of activated NK cells, exhibited a negative correlation. Furthermore, the relationship between risk scores and the ESCC microenvironment was assessed using the Estimate algorithm, and the findings indicated that there is a substantial positive link between stromal and immune scores and the tumor microenvironment in high-risk patients, which often comprises a larger number of immune cells and stromal cell components (**Figure 4E**).

### 3.6 single-cell analysis of coagulation feature genes in ESCC

**Figure 5:**
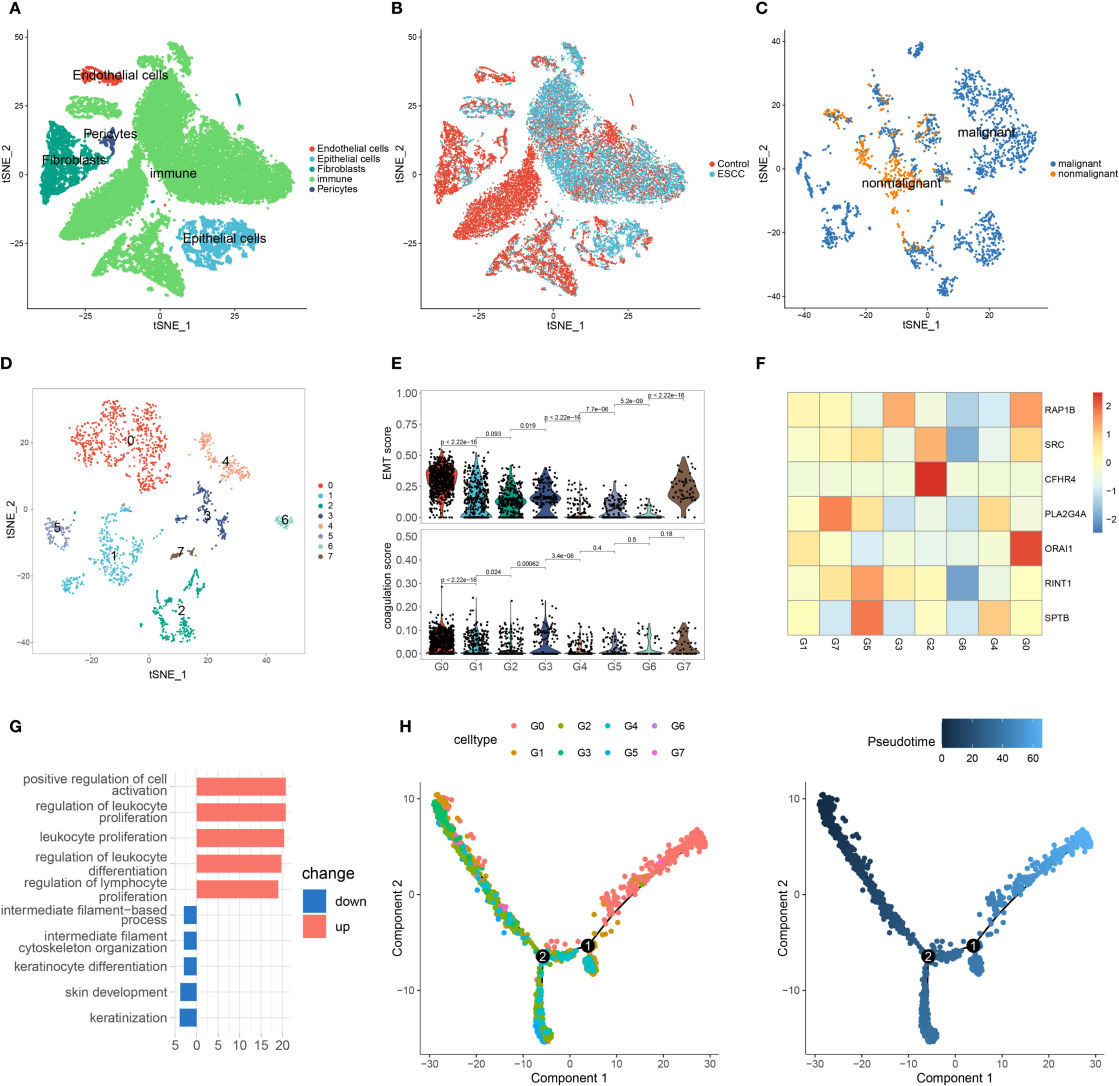
Single-cell analysis of ESCC. **(A)** t-SNE plot showing the five cell types after clustering. **(B)** t-SNE plot of cells from different sample sources. **(C)** t-SNE plot of epithelial cell clustering based on malignant score. **(D)** After extracting malignant epithelial cells, they were further divided into eight subgroups. **(E)** EMT and coagulation scores of different malignant epithelial cell subgroups. **(F)** Expression of coagulation feature genes in malignant epithelial cell subgroups. **(G)** Enriched classical pathways in G0 cells based on differentially expressed genes across subgroups. **(H)** Pseudotime analysis of malignant epithelial cell subgroups.

We conducted a detailed analysis using single-cell sequencing data from GSE196756. After quality control, we identified five major cell types: endothelial cells, epithelial cells, fibroblasts, pericytes, and immune cells (**Figure 5A**, **Supplementary Figure S4A**). Epithelial and immune cells were more abundant in ESCC tissues, indicating tumor microenvironment remodeling (**Figure 5B**). Since ESCC originates from epithelial cells, we focused on epithelial subpopulations, performed clustering, and calculated malignancy scores using differential genes from the GSE53622 and GSE53624 datasets. This classified epithelial cells into malignant and non-malignant groups, with malignant cells being the majority (**Figure 5C**, **Supplementary Figure S4B**).

Upon further analysis of malignant epithelial cells, we identified eight distinct subpopulations, designated as G0 through G7 (**Figure 5D**). To investigate their characteristics, we calculated epithelial–mesenchymal transition (EMT) scores using a previously reported ESCC-specific EMT gene set, and derived coagulation scores based on the expression of key coagulation-related genes. Notably, the G0 cluster exhibited the highest levels of both EMT and coagulation scores (**Figure 5E**). We then assessed gene expression patterns across subpopulations, and heatmap analysis revealed that G0 cells showed the highest expression levels of coagulation-related genes (**Figure 5F**). These findings suggest a potential association between coagulation-related pathways and the EMT process in ESCC.

To investigate functional characteristics, we compared G0 and G1 subpopulations through differential gene analysis and GO enrichment. Upregulated genes in G0 cells were enriched in immune response and cell proliferation pathways, possibly linked to immune escape or tumor growth in ESCC (**Figure 5G**). Downregulated pathways involved cytoskeleton regulation and keratinocyte differentiation, which may impact tumor cell migration and differentiation. In pseudotime analysis, G0 cells were located at the end of the trajectory (**Figure 5H** and **Supplementary Figure S4C**), suggesting a more advanced, invasive tumor stage.

**Figure 6:**
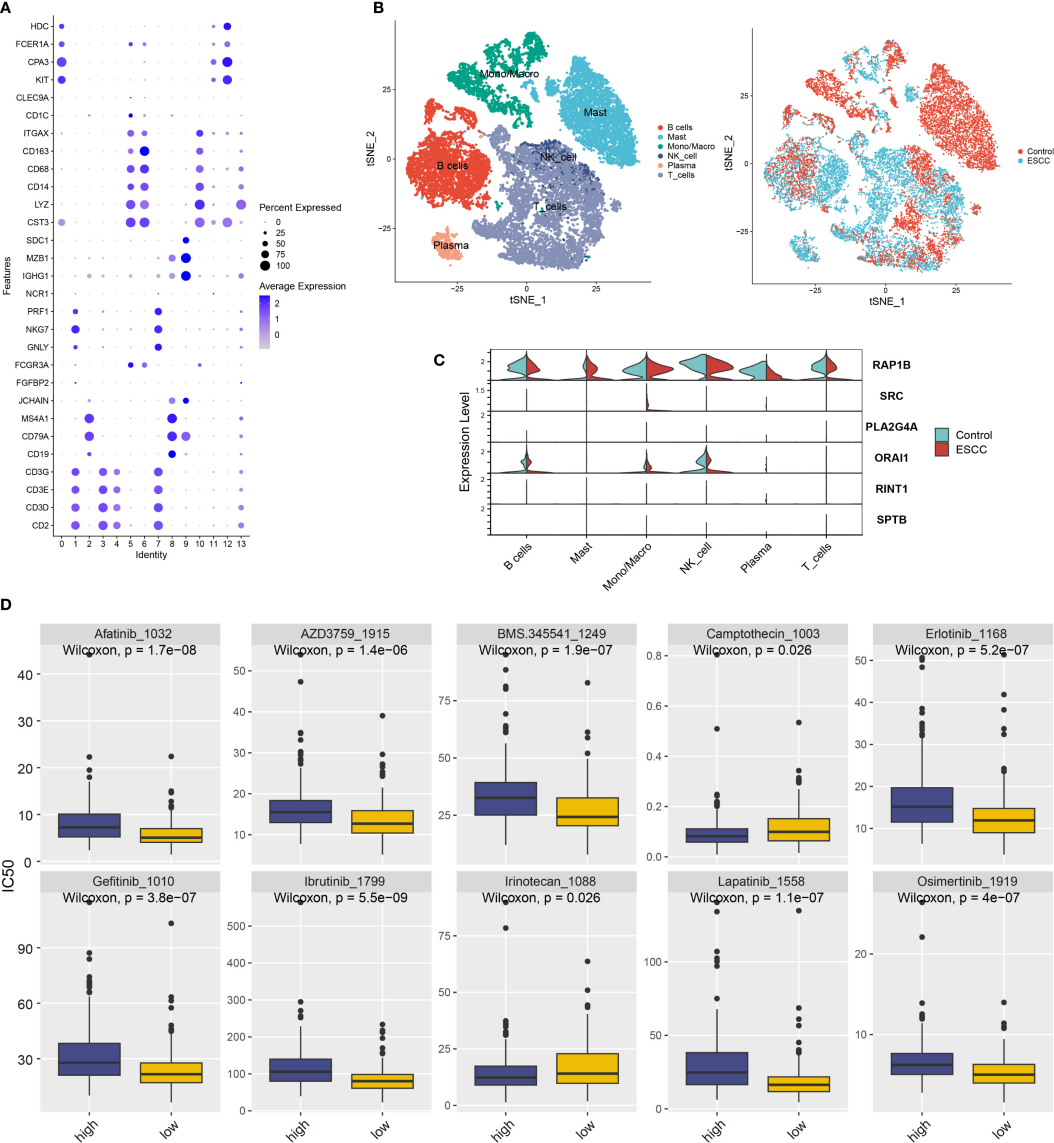
Coagulation feature genes analysis in ESCC immune cell subgroups and drug sensitivity. **(A)** Classification of 14 immune cell subgroups based on immune cell marker genes. **(B)** t-SNE plot showing six immune cell types after clustering, with cells from different sample sources included. **(C)** Violin plot showing the expression of coagulation feature genes across different immune cell types. **(D)** Prediction of IC50 values for various anticancer drugs based on risk scores.

Lastly, we analyzed immune cells by identifying six subpopulations and found that T cells were predominant (**Figures 6A, B**, **Supplementary Figure S4D**). Coagulation gene expression analysis revealed high expression of RAP1B and ORAI1 in various immune cell types (**Figure 6C**).

### 3.7 Drug sensitivity analysis of coagulation feature genes

We used the “oncoPredict” program to do a medication sensitivity study in order to investigate possible treatment options for ESCC patients. We discovered notable variations in medication responses between high-risk and low-risk groups based on the model categories (**Figure 6D**). The high-risk group was more sensitive to irinotecan and camptothecin, whereas the low-risk group was more sensitive to afatinib, erlotinib, and gefitinib. Some of these drugs are already in clinical use and have demonstrated good efficacy, while others require further validation. Our findings provide valuable insights for identifying potential drugs for ESCC treatment in clinical practice.

### 3.8 Identification of the key coagulation gene RINT1 and pan-cancer analysis

**Figure 7:**
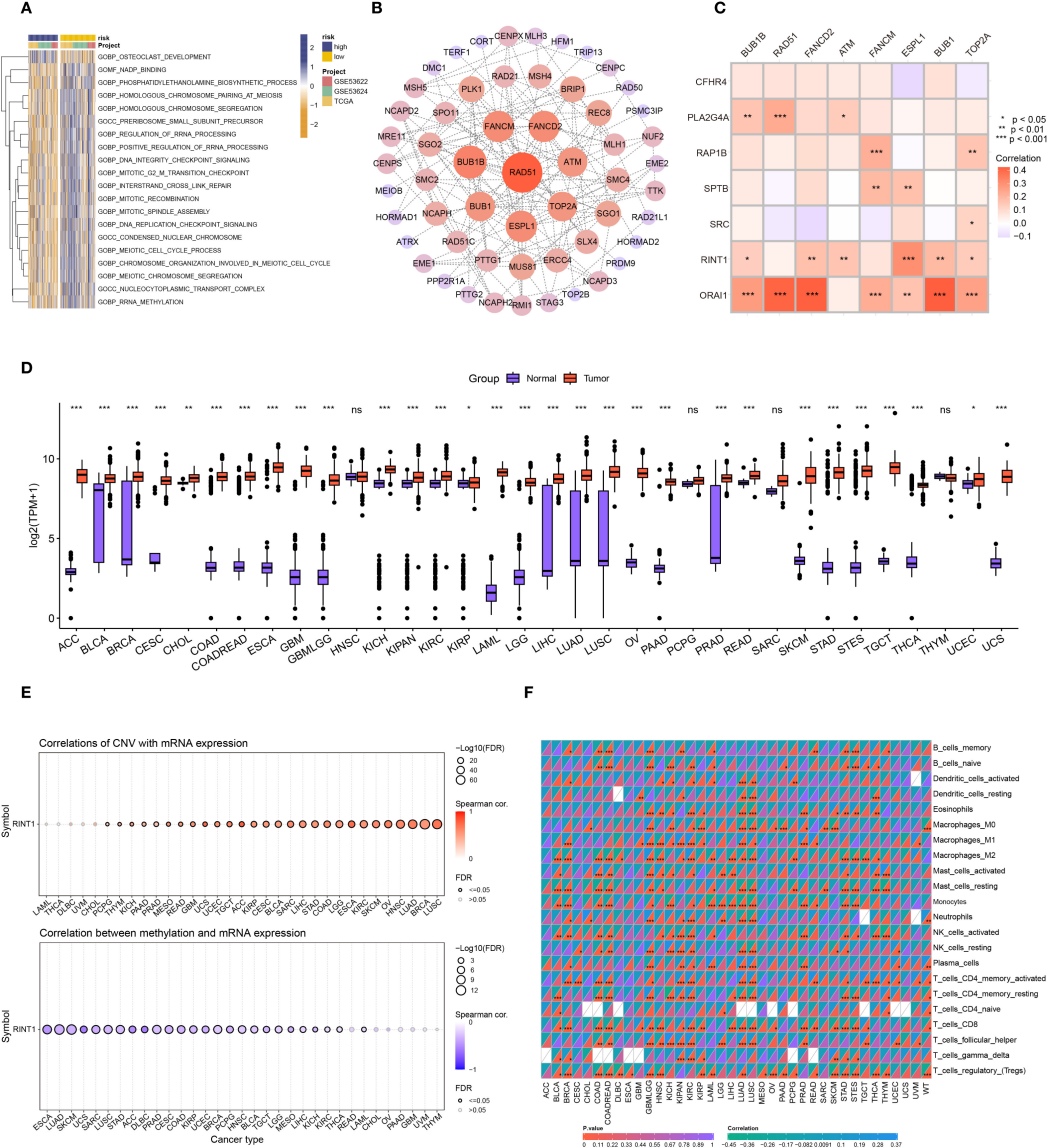
Pan-cancer analysis of RINT1. **(A)** GSVA analysis heatmap for patients in high and low-risk groups. **(B)** Protein-protein interaction (PPI) network of genes from the top 5 significantly enriched pathways identified by GSVA. **(C)** Correlation heatmap showing the relationship between coagulation-related genes and the 8 genes most significantly correlated based on PPI. **(D)** Differential analysis of RINT1 expression between tumor and normal patients in TCGA and GTEx databases. **(E)** Correlation of CNV and DNA methylation with RINT1 expression across different cancers. **(F)** Immune infiltration analysis of RINT1 in pan-cancer studies.

The top 20 differentially enriched pathways in high- and low-risk groups were investigated in order to more accurately assess the connection between risk stratification and GSVA scores in ESCC. The findings showed that there were notable variations between the two groups in a number of pathways, with the high-risk group exhibiting reduced enrichment in meiosis, rRNA processing, and rRNA methylation (**Figure 7A**). To identify key genes associated with coagulation, we extracted relevant genes from the top five enriched GSVA pathways and subsequently identified eight key genes through protein-protein interaction (PPI) network analysis (**Figure 7B**). Gene correlation analysis revealed strong associations between ORAI1 and RINT1 with genes in these key pathways (**Figure 7C**), suggesting that they may play a central role in coagulation feature gene networks.

ORAI1, a critical calcium ion channel protein, regulates intracellular calcium balance and various cellular functions. Prior research has demonstrated the involvement of ORAI1 in the development of ESCC, potentially influencing tumor proliferation and metastasis. As a coagulation-related gene, ORAI1 holds significant biological relevance in cancer progression. RINT1, which encodes a protein involved in cell cycle regulation and DNA damage repair, has been studied in the context of pancreatic and breast cancers (41, 42). However, its role in ESCC remains underexplored. Therefore, RINT1 was chosen as a crucial coagulation gene for additional research in this work, with plans to explore its possible processes in ESCC.

To comprehensively assess RINT1’s role in various cancers, we conducted a pan-cancer analysis. Using data from the TCGA and GTEx databases, we examined RINT1 expression across multiple cancer types. Boxplots indicated significant expression differences of RINT1 in most cancer types(**Figure 7D**). Further analysis revealed that RINT1 expression and gene copy number variation (CNV) were positively correlated, but that DNA methylation levels were negatively correlated (**Figure 7E**). This positive correlation suggests that increased CNV may lead to upregulation of RINT1 expression, promoting cancer cell proliferation and growth. Conversely, DNA methylation is typically associated with gene silencing, and its negative correlation with RINT1 expression suggests that lower methylation levels in cancer samples may enhance RINT1 gene expression.

Using the CIBERSORT program, we also conducted a pan-cancer immune infiltration investigation of RINT1. According to the findings (**Figure 7F**) RINT1 expression is strongly correlated with immune cell types in a number of malignancies, such as head and neck squamous cell carcinoma, lung cancer, gastric cancer, renal cell carcinoma, and glioblastoma. These results imply that RINT1 may affect the initiation and progression of cancer by modifying immune responses in the tumor microenvironment.

Overall, our findings provide further evidence of the possible function of the coagulation gene RINT1 in various cancers, offering a theoretical basis for future research into its specific mechanisms in cancer development and progression.

### 3.9 Analyzing the multi-layered regulatory network of RINT1 and its *in vitro* expression validation in ESCC

**Figure 8:**
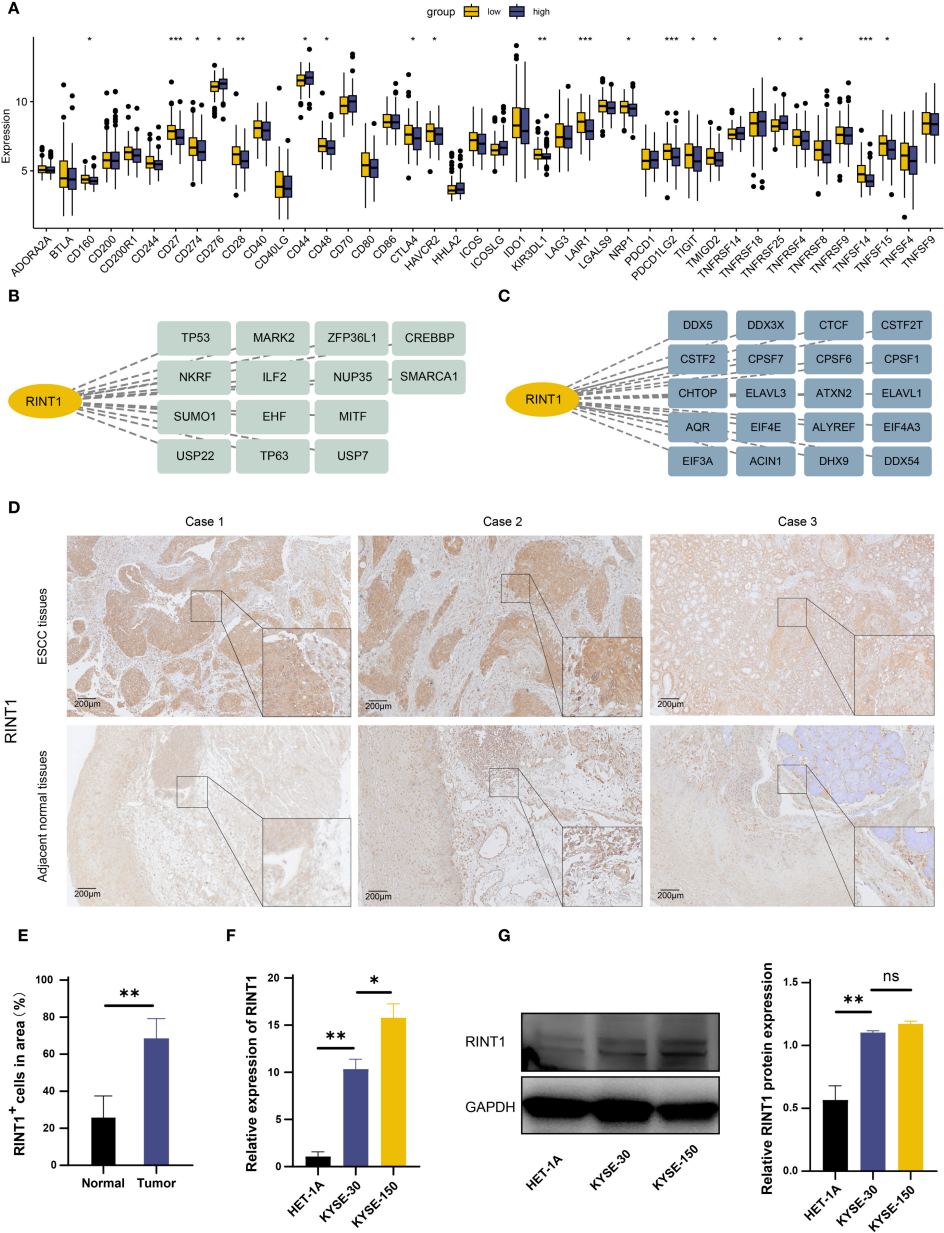
Functional identification of RINT1 and *in vitro* validation of its expression levels. **(A)** Differential expression of immune checkpoints between high and low RINT1 expression groups. **(B)** Analysis of RINT1-associated transcription factors. **(C)** Analysis of RINT1-associated RNA binding proteins. **(D)** Immunohistochemical analysis demonstrated a significant upregulation of RINT1 expression in ESCC tissues. **(E)** Quantitative analysis of RINT1 immunohistochemistry results. **(F)** qRT-PCR results confirmed the differential expression levels of RINT1 between normal esophageal cell lines (HET-1A) and ESCC cell lines (KYSE-3O and KYSE-150). **(G)** Western blot analysis further validated the high expression of RINT1 in ESCC cell lines. *p-value < 0.05, **p-value < 0.01, ***p-value < 0.001.

We examined RINT1’s interaction with immunological checkpoint inhibition (ICB) molecules in order to learn more about its function in tumor growth and the immune milieu. RINT1 expression and other immunological checkpoint molecules were shown to be significantly correlated (**Figure 8A**). Specifically, low expression of immunological checkpoints such as PD-L1 (CD274), PD-L2 (PDCD1LG2), and CTLA-4 (CTLA4) was negatively correlated with high expression of RINT1. This implies that RINT1 may regulate immune checkpoint expression, impacting tumor immune evasion strategies and being a crucial component of the tumor IME.

Transcription factors are essential for the regulation of gene expression. **Figure 8B** shows possible upstream transcription factors of RINT1; alterations in transcription factors can result in aberrant expression of tumor-related genes, which further promotes tumor cell migration, metastasis, and proliferation; RNA-binding proteins (RBPs) are important for post-transcriptional regulation of gene expression; RINT1 may interact with different RBPs, including DDX3X, CTCF, and ELAVL1 (**Figure 8C**), which regulate post-transcriptional modifications of tumor-related genes and impact tumor cell growth, invasiveness, and immune evasion.

The role of RINT1 in tumor progression has been preliminarily confirmed through the analyses above. To further examine its expression and function in ESCC, we compared RINT1 levels in ESCC tissues and adjacent normal tissues using immunohistochemistry. According to the results, ESCC tissues had significantly higher RINT1 expression. (**Figures 8D, E**). We then performed qRT-PCR in ESCC cell lines (KYSE-30 and KYSE-150) and normal esophageal epithelial cells (HET-1A). The qRT-PCR results indicated significantly higher RINT1 expression in the ESCC cell lines compared to the normal cells (**Figure 8F**). Western blot analysis confirmed this finding, showing a substantial increase in RINT1 protein expression in the ESCC cell lines (**Figure 8G**). Overall, the elevated expression of RINT1 in ESCC suggests its potential involvement in tumorigenesis and progression, highlighting RINT1 as a possible biomarker for the diagnosis and treatment of ESCC.

## 4 Discussion

Numerous studies have examined the strong correlation between coagulation malfunction and tumor progression (43), and CAT and a hypercoagulable condition have been found to be potential contributors to the development of certain malignancies. Multiple genes participate in ESCC, a multifactorial disease impacted by intricate interactions between genetic and environmental variables (44, 45). Multiple studies have demonstrated the critical roles that different coagulation function markers play in the development of ESCC, pointing to a possible connection between coagulation function and the initiation and progression of ESCC (46).

Genes are fundamental units carrying genetic information within organisms, encoding proteins that determine both external traits and internal metabolic processes (47). Genes involved in the coagulation pathway regulate coagulation function, yet previous studies have not extensively explored the genetic aspects of the connection between coagulation and ESCC. Our research innovatively explored the mechanisms and potential significance of coagulation-related genes in ESCC using bioinformatics methods. We comprehensively analyzed the biological functions, prognostic value, clinical relevance, immune implications, tumor microenvironment interactions, and drug sensitivity of these genes, demonstrating their pivotal role in ESCC through multi-omics data.

In recent years, machine learning methods have increasingly contributed to cancer research by identifying genes relevant to disease mechanisms (48). We employed a variety of machine learning techniques and compared their average c-index, revealing that the RSF method achieved the highest performance. RSF, a robust algorithm for survival analysis, effectively handles missing data and identifies critical features associated with survival time, demonstrating significant practical utility in real-world applications (49). We used RSF-selected genes in multivariate Cox regression analysis to find feature genes that were highly correlated with patient survival. RAP1B, SRC, CFHR4, PLA2G4A, ORAI1, RINT1, and SPTB are the seven coagulation-related genes that were discovered using this method.

In our study, we established a prognostic model aimed at predicting the clinical outcomes of ESCC patients, drawing upon the expression of coagulation feature genes. This model was verified by various kinds of methods, proving its high predictive power. This suggests the potential application of the model in personalized treatment for ESCC. Additionally, we explored the mechanisms underlying these coagulation feature genes in ESCC. The results indicate that these genes regulate tumor immune evasion and progression by affecting key biological processes such as immune cell infiltration, tumor microenvironment, meiosis, rRNA processing, and DNA methylation. Notably, while activated NK cell expression was markedly increased in high-risk patients, other important immune cells such as plasma cells, follicular helper T cells (Tfh), and activated mast cells were inhibited. Tumor cell immune evasion may be facilitated by this inhibition. Furthermore, higher stromal, immune, and ESTIMATE scores were associated with high-risk patients, suggesting a tumor microenvironment that is both more complex and heterogeneous. These findings may reflect the genetic instability inherent in tumor cells, which could foster both cancer progression and resistance to treatment (50).

To further understand the complex cellular landscape of the ESCC microenvironment, we employed single-cell sequencing data to conduct a thorough investigation of cellular heterogeneity. Our analysis revealed that epithelial cells derived from ESCC tissues had significantly higher malignancy scores and could be further subdivided into multiple functional subpopulations. Among these, the G0 subpopulation not only exhibited the highest EMT score but also showed high expression of key coagulation genes, suggesting a potential synergistic relationship between the activation of coagulation pathways and the EMT process. This finding further supports the idea that coagulation contributes to tumor progression and enhanced invasiveness. Immune cell analysis indicated that T cells predominated in the tumor microenvironment, with coagulation-related genes such as RAP1B and ORAI1 showing high expression across various immune cell types. This implies that by altering immune cell functions, coagulation-related genes may also have an impact on the ESCC IME.

Our study identifies RINT1 as a key coagulation-related gene with potential implications in ESCC. Previous studies have shown that RINT1 is localized to the Golgi apparatus, centrosome, and endoplasmic reticulum, and is involved in maintaining mitotic stability. In heterozygous RINT1-deficient mice, approximately 81% spontaneously developed multiple tumors, suggesting its potential tumor-suppressive role (51). However, RINT1 has also demonstrated oncogenic properties. For instance, in glioblastoma (GBM), overexpression of RINT1 promotes cellular transformation and tumor formation (52); in pancreatic ductal adenocarcinoma (PDAC), RINT1 contributes to tumor progression by regulating DNA repair, cell cycle, ER-Golgi homeostasis, SUMOylation, and apoptosis (41). These findings indicate that RINT1 exhibits functional heterogeneity across different tumor types. In our study, high RINT1 expression was associated with better prognosis in the prognostic model, yet it was significantly upregulated in ESCC tissues compared to normal controls, suggesting its involvement in tumor development. Further immune analysis revealed that RINT1 expression was negatively correlated with immune checkpoint molecules PD-L1 and CTLA-4, which are typically linked to immune suppression and tumor immune evasion (53). RINT1 expression also showed a positive correlation with immune-activating cell subsets such as T follicular helper cells and memory B cells, implying a potential association between RINT1 overexpression and enhanced antitumor immunity. These findings suggest that RINT1 may not serve as a purely oncogenic or tumor-suppressive factor in ESCC, and its functional role may be more complex. Collectively, RINT1 may exert multifaceted, context-dependent functions in ESCC by influencing both tumor biology and the immune microenvironment. Future studies integrating functional assays, cell models, and clinical subgroup analyses are warranted to elucidate its molecular mechanisms and therapeutic potential.

This study provides valuable insights into the role of coagulation-related genes in ESCC and the development of a prognostic model, however, some inevitable limitations remain. First, The biological functions and clinical relevance of these genes have been analyzed, but the molecular mechanisms regulating coagulation activity in ESCC remain unclear. A deeper exploration of these mechanisms could help clarify how coagulation influences tumor progression and reveal potential therapeutic opportunities. Second, The prognostic model has demonstrated good predictive potential, yet its reliability needs to be tested on larger, multi-center clinical datasets to confirm its broader applicability. Furthermore, The connection between coagulation-related genes and tumor immune regulation also requires more detailed study. These genes may have a more complex role in shaping the tumor microenvironment than current findings suggest. Additional experimental validation and expanded clinical analyses are essential to evaluate their potential value as therapeutic targets. Overall, this study lays a foundation for future research on coagulation mechanisms in ESCC and holds promise for advancing early diagnosis, risk stratification, and personalized treatment strategies.

## 5 Conclusion

Our study offers a comprehensive analysis of the expression patterns of coagulation-related genes in ESCC, highlighting their potential involvement in tumor progression. Through our investigation, we identified coagulation feature genes and established a prognostic model based on these markers, demonstrating its clinical reliability. Further examination suggests that these genes may influence tumor development by modulating the TME, immune responses, and various biological pathways. The identification of RINT1 as a potential prognostic biomarker and candidate for future therapeutic investigation is a notable finding that warrants deeper exploration in subsequent studies. While additional experiments and clinical validation are certainly necessary to confirm these findings, our results seem to provide valuable insights into the role of coagulation-related genes in ESCC. These discoveries provide a foundation for future development of more personalized and targeted treatment strategies, which may ultimately improve clinical outcomes and prognosis for ESCC patients.

## Data Availability

The original contributions presented in the study are included in the article/**Supplementary Material**. Further inquiries can be directed to the corresponding author.
