# Supplementary material for: Unraveling the role of coagulation-related genes in esophageal squamous cell carcinoma: development of a prognostic model and exploration of potential clinical significance
Source: Front Oncol. 2025 Oct 7;15:1573279. doi: 10.3389/fonc.2025.1573279 (PMC12537425; doi:10.3389/fonc.2025.1573279)
Supplement: Supplementary file 1 [file Table1.docx]

Supplementary Material


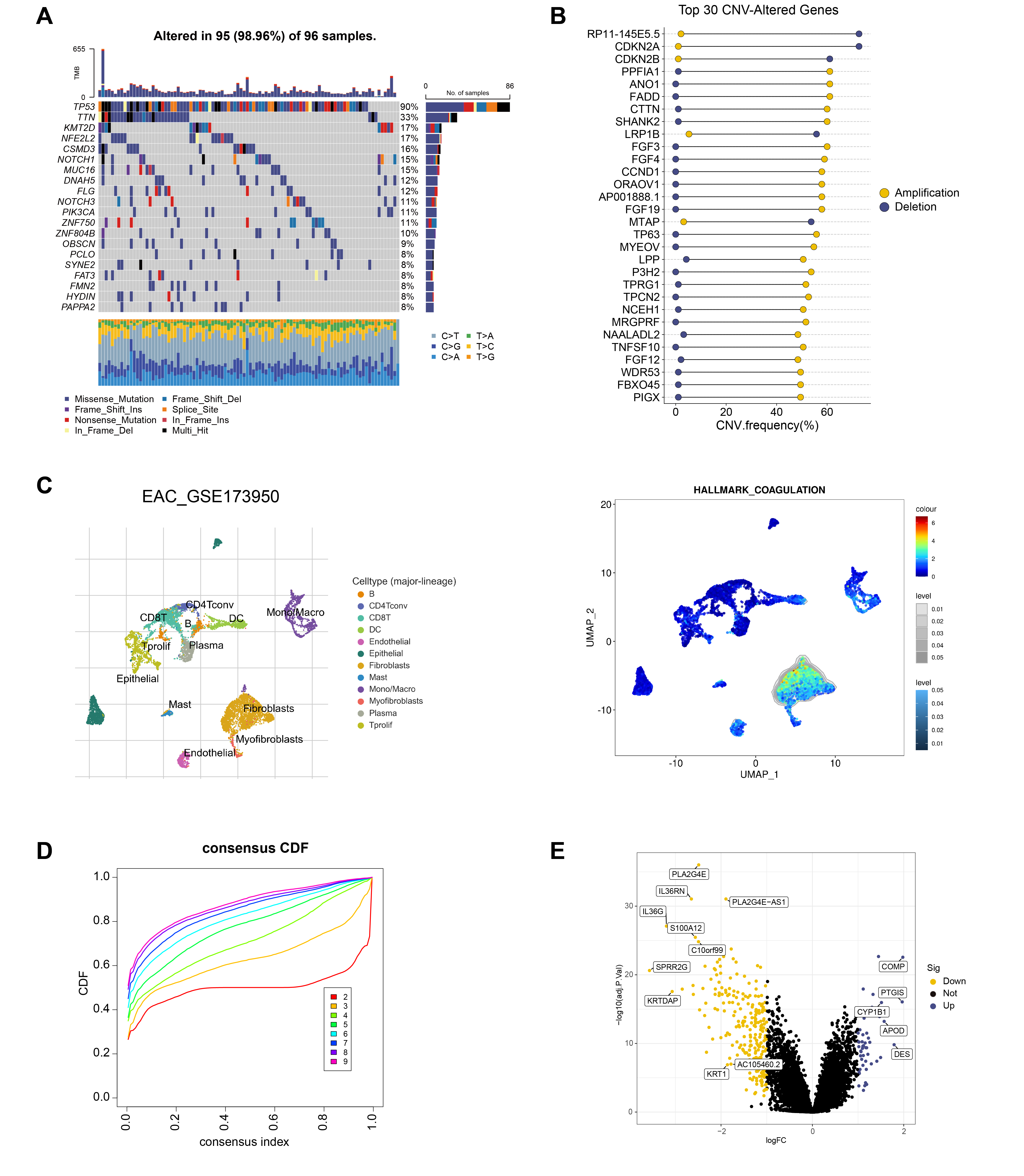
 **Supplementary Figure. S1**

Figure S1. (A) Oncoplot of somatic mutations in all sequenced genes in the TCGA-ESCA cohort for ESCC.

(B) Dumbbell plot of the top 30 genes with CNV alterations among all sequenced genes in ESCC.

(C) Enrichment Scores of the Coagulation Pathway Across Different Cell Types in the GSE173950 Dataset Using GSEA.

(D) Cumulative distribution function (CDF) plot of clustering consistency for coagulation-related genes.

(E) Volcano plot of differential gene expression analysis of ESCC sequencing genes based on the two subtypes.

**Supplementary Figure. S2**


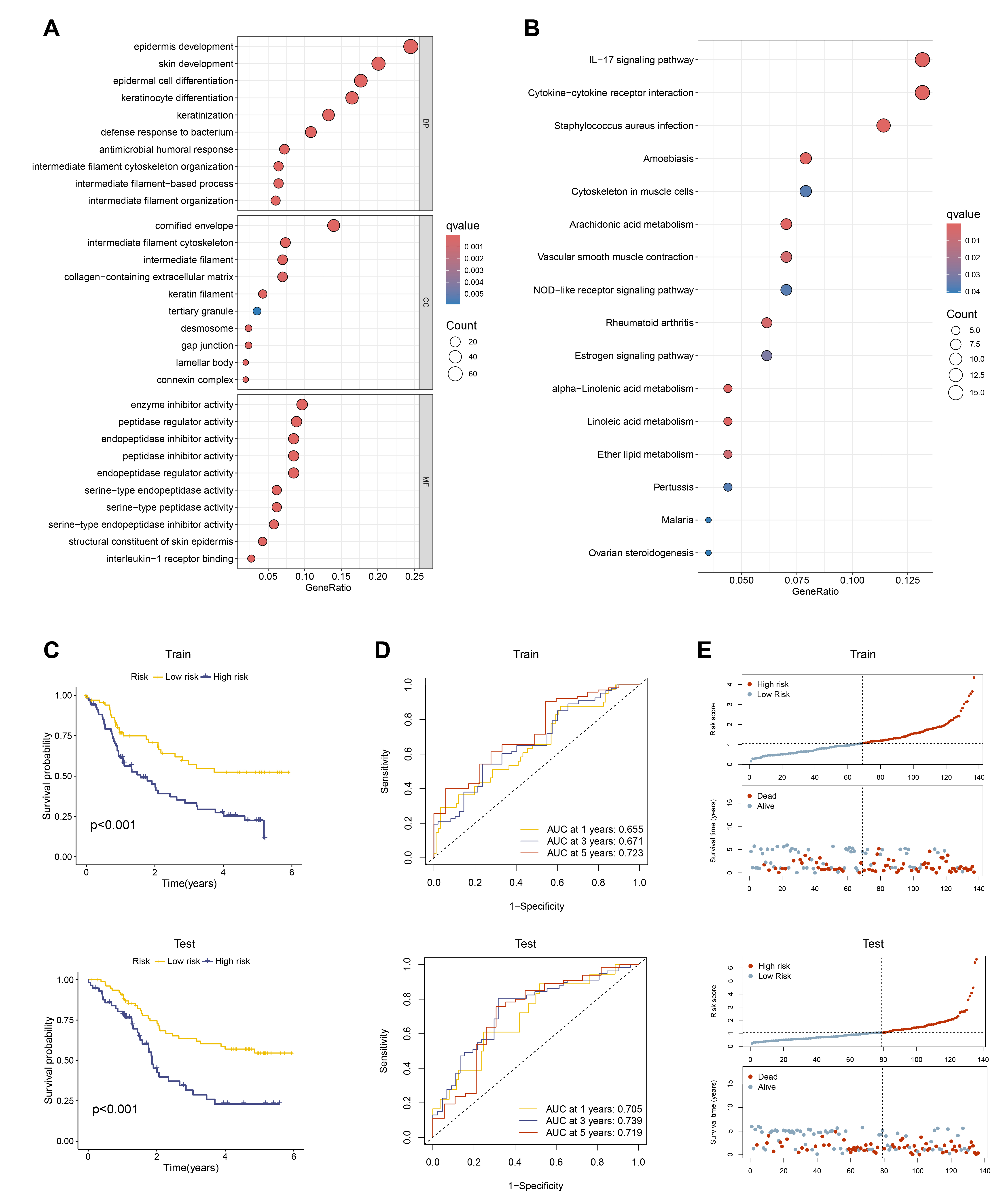


Figure S2. (A) Visualization of GO analysisfor differential genes.

(B) Visualization of KEGG analysis for differential genes.

(C) Survival analysis of high-risk and low-risk patient groups in the traini and test cohort.

(D) ROC curves and corresponding AUC values analyzed for patients stratified into high- and low-risk groups in the train and test cohort at 1, 3, and 5 years.

(E) Risk curves for high-risk and low-risk patient groups in the train and test cohort.

**Supplementary Figure. S3**


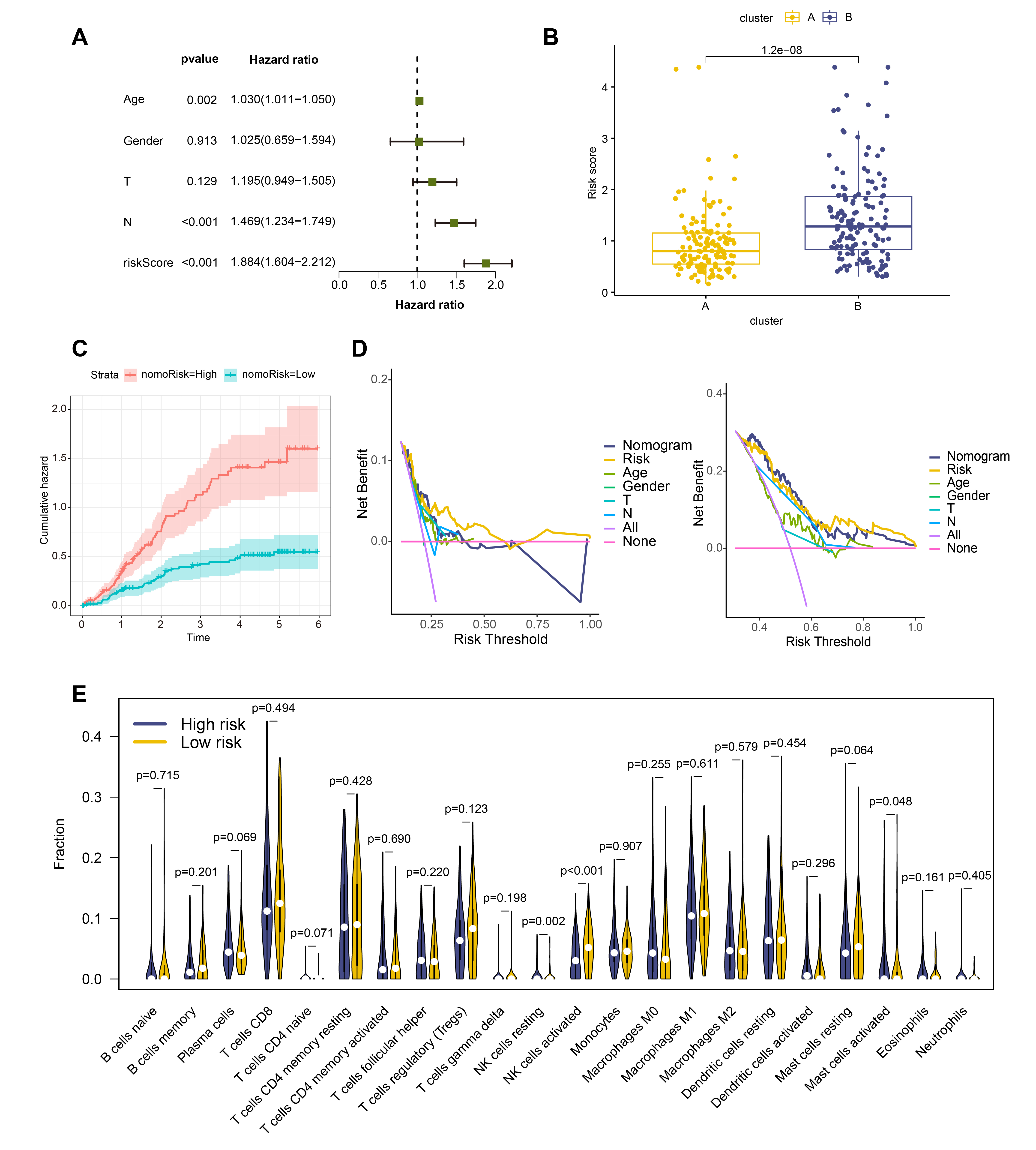
Figure S3. (A) Univariate Cox regression analysis of risk scores with age, gender, T stage, and N stage.

(B) Relationship between two subtypes and risk scores

(C) Cumulative risk curve for patients in high and low-risk groups.

(D) Decision curve analysis (DCA) at 1 and 3 year.

(E) Analysis of immune cell differences in high and low-risk group patients.


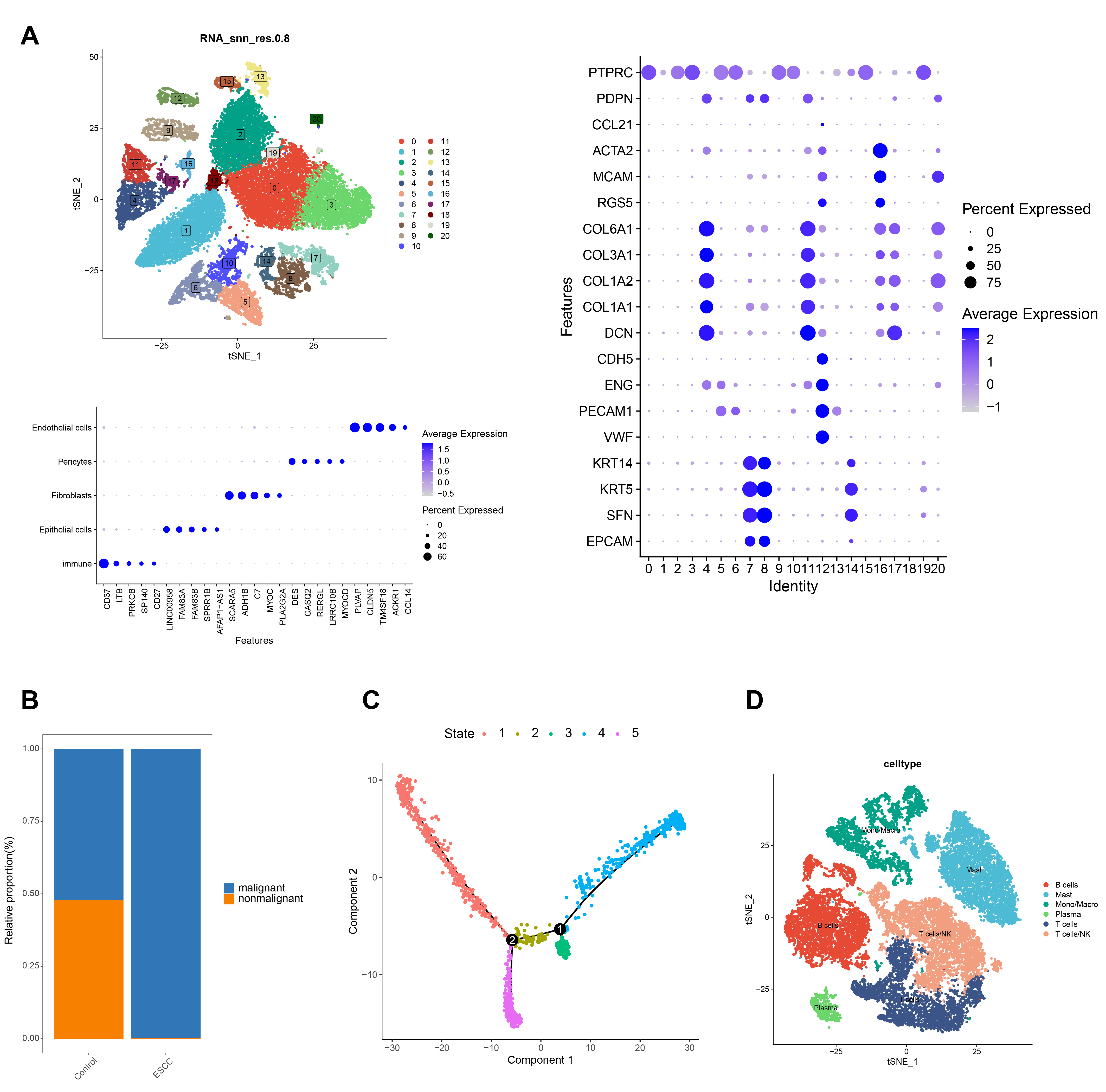
**Supplementary Figure. S3**

Figure S4.(A) Identifying 21 clusters at a resolution of 0.8, with preliminary identification of subgroups based on marker genes. The top 5 most highly expressed genes in each subgroup are displayed.(B) Proportions of malignant and non-malignant cells in epithelial cells from different sample sources.(C) Pseudotime analysis reveals the presence of 5 distinct cell states.(D) t-SNE plot of immune cells after preliminary clustering based on marker genes.
